# A trespasser from a foreign land? A case report of primary mucosal leishmaniasis

**DOI:** 10.1186/s12879-022-07169-w

**Published:** 2022-03-03

**Authors:** Yoram Fleissig, Mary Dan-Gur, Ayelet Michael-Gayego, Alexander Maly, Rami Tabib, Charles L. Jaffe, Maya Korem

**Affiliations:** 1 Faculty of Dental Medicine, Hebrew University of Jerusalem, PO Box 12272, Jerusalem, 9112102 Israel; 2 Department of Oral and Maxillofacial Surgery, Hadassah Medical Center, Jerusalem, Israel; 3 Faculty of Medicine, Hebrew University of Jerusalem, Jerusalem, Israel; 4 National Center for Leishmaniasis, Kuvin Center for Study of Tropical and Infectious Diseases, Hadassah Medical Center, Jerusalem, Israel; 5 Department of Clinical Microbiology and Infectious Diseases, Hadassah Medical Center, Jerusalem, Israel; 6 Department of Pathology, Hadassah Medical Center, Jerusalem, Israel

**Keywords:** Mucosal, Leishmaniasis, Leishmania donovani, Case report

## Abstract

**Background:**

We report a clinically challenging and unusual case of *L. donovani* oral mucosal leishmaniasis.

**Case presentation:**

Israeli resident with a former travel to central and North Africa, with no documented or prior cutaneous lesions presented with oral lesions of the maxillary gingiva and the upper lip. A delay in diagnosis and treatment have led to progression of the maxillary gingival lesions towards the hard palatal and the soft palate that could have potentially compromised the upper airway.

**Conclusions:**

This case highlights the importance of early diagnosis of leishmaniasis in patients with oral lesions and the laboratory workup necessary to appropriately characterize and treat the disease.

## Background

Leishmaniasis is a parasitic disease transmitted by phlebotomine sand flies (*Phlebotomus* in the Old World and *Lutzomyia* in the New World) which is caused by ~ 25 different species in about 100 endemic countries spanning both tropical and temperate regions. New areas are at risk for leishmaniasis for diverse reasons such as global warming, forest destruction and the use of immunosuppressants for chronic diseases. Following a bite, promastigotes are phagocytized by mononuclear phagocytic cells and transform into amastigotes, which multiply by simple division and proceed to infect other mononuclear phagocytic cells. Clinical manifestations are divided into cutaneous, mucosal and visceral (kala-azar) forms, with the latter involving blood, bone marrow and inner organs. Mucosal leishmaniasis (ML) occurs mainly in the New World and involves upper respiratory tract, nose and oral cavity. It is caused by the *Viannia* subgenus, primarily *L. (V.) braziliensis* [1]. Typically, ML appears days to years, sometimes decades, after the initial cutaneous lesion with the risk factors for ML including large or multiple cutaneous lesions and long-standing skin lesions. Nevertheless, about 30% of the patients with mucosal lesions do not recall a primary cutaneous lesion [1, 2]. ML is a progressive disease that without proper treatment will destroy facial structures and compromise the upper airway.

Outside the South American continent, particularly in the Mediterranean basin, *L. infantum* has been associated with ML and can be accompanied or preceded by either cutaneous leishmaniasis (CL) or visceral leishmaniasis (VL), although ML can be the only documented pathological condition. *L. tropica* and *L. major* rarely cause ML. ML caused by *L. donovani*, a common agent of VL, is a rare but increasingly reported entity from the endemic regions, mainly India, Sudan and Sri Lanka [3]. The mechanism by which the mucosal involvement occurs in *L. donovani* infection is unclear and may resemble that of *L. infantum* ML which is presumed to disseminate through the lymphatics or hematogeneously. Oral ML especially by *L. donovani* can cause severe damage, such as tooth loss and severe respiratory obstruction. Lesions are usually described as whitish, reddish, violaceous nodules or polypoid masses developing in a swollen mucosa and contain abundant *Leishmania* bodies. Little is known about the pathogenesis of ML caused by species other than *L. braziliensis*. Experimental models show that ML is associated with uncontrolled inflammation and that ML develops in the presence of insufficient immune response in early staged of CL [3]. It was also suggested that some *Leishmania* strains such as *L. infantum* had acquired the capability to live in mucous membranes through variable sensitivity to elevated temperature [4].

CL has long been endemic in Israel and is caused mainly by *L. major* in the southern part of the country and by *L. tropica* in the Northern part. Human VL caused by *L. infantum* is reported in central and North Israel. ML in Israel had been described so far only in travelers returning from endemic areas for *L. braziliensis* [5].

## Case presentation

**Fig. 1:**
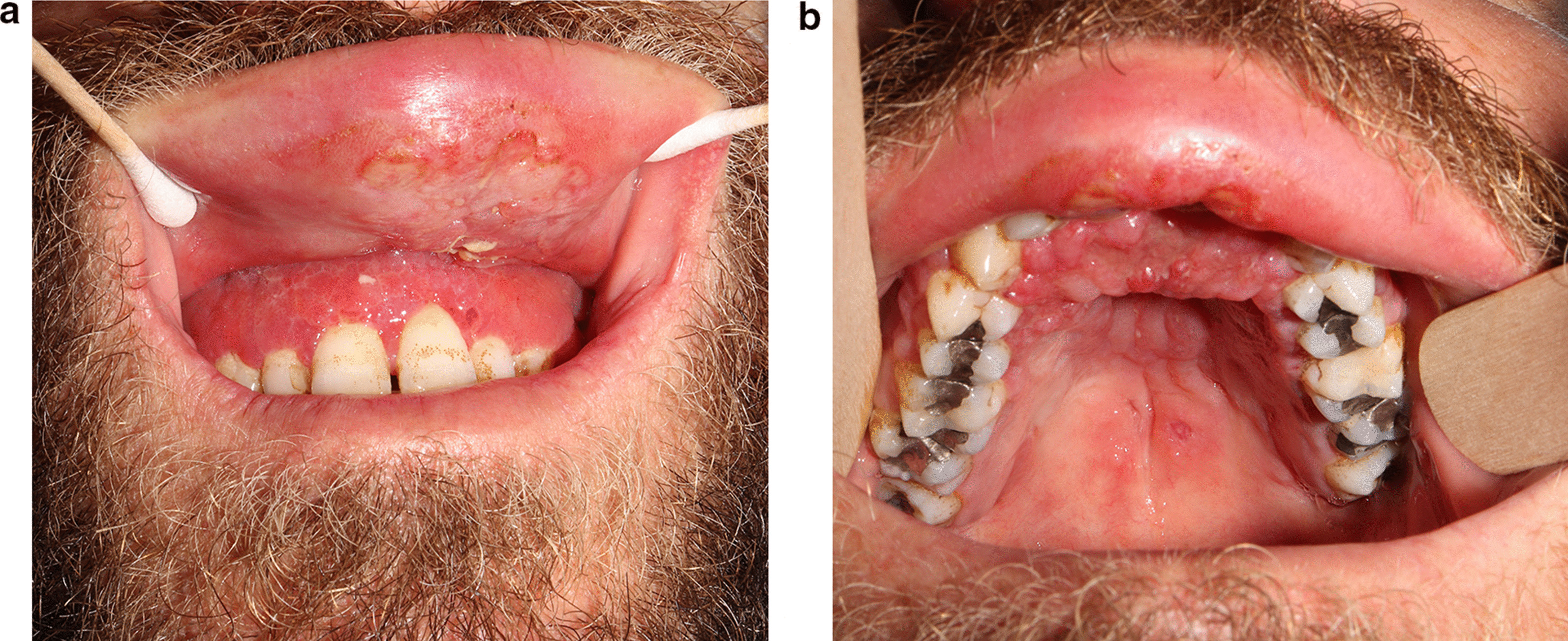
Maxillary gingival hyperplasia and upper lip polypoid swelling and superficial ulcerations in its inner aspect (**a**). progression of the lesions and involvement of the palatal gingiva and hard palate, and ulcerations in the soft palate (**b**)

**Fig. 2:**
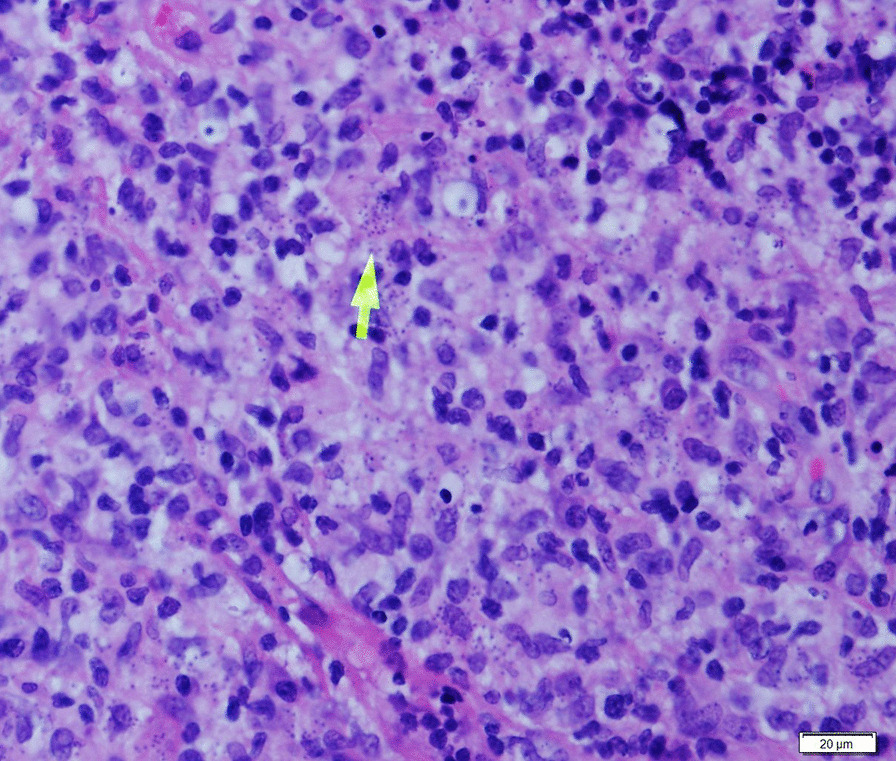
Histopathological examination of biopsy of oral lesion showing amastigotes forms of *Leishmania* within macrophages (arrow) in Hematoxilin-Eosin stained section (×600). Microscopy images were captured using Olympus BX51 light microscope, Olympus DP72 camera and Olympus cellSens imaging software

A 30-year-old healthy non-smoker male presented to the Periodontology outpatient clinic. His chief complaint was upper lip swelling over the last few months, desquamating of the maxillary gingiva, and discomfort, especially when chewing. Clinical examination revealed maxillary gingival hyperplasia between the upper canines, poor oral hygiene and dental calculus, with no tooth mobility or attachment loss. Dental X-ray did not show abnormality. Deep scaling and root planning conjoined with antibiotic treatment (metronidazole for 7 days) did not improve the gingiva appearance, and therefore he was referred to the Oral and Maxillofacial outpatient clinic. Physical exam revealed maxillary gingival hyperplasia and upper lip polypoid swelling and superficial ulcerations in its inner aspect (Fig. 1a). Also, progression of the lesions and involvement of the palatal gingiva and hard palate, and ulcerations in the soft palate were found (Fig. 1b). No facialis or other head and neck neurologic deficit were found. Nasal exam showed few lesions in the columella region and the Kiesselbach mucosa. The patient denied fever and night sweats, and reported a weight loss of about 8 Kg. He also denied getting any immunosuppressive drugs. Chest X-ray was normal. Abdominal ultrasound showed mild splenomegaly of 15.4 cm (the upper normal limit is 14 cm and positively correlated with body height) [6], complete blood count and liver function tests were normal. Differential diagnosis included granulomatous disease (such as Granulomatosis with polyangiitis and orofacial granulomatosis), lymphoproliferative disease, myeloid malignancy (such as acute myeloid leukemia) and pyogenic granuloma. Incisional biopsies were taken from the inner aspect of the upper lip and the palatal gingiva. Hematoxylin and eosin histopathology sections revealed dense infiltrate of macrophages with abundant *Leismania* organisms. In the background plasma cells, lymphocytes and non-caseating granulomas (Fig. 2). Wright-stained smear of the tissue revealed macrophages with multiple cytoplasmic oval amastigotes. Thorough anamnesis revealed that the patient works as a date farmer in an endemic region for cutaneous leishmaniasis in Israel, and spent several months in North Africa and Zambia 4 years ago. Nevertheless, the patient did not recall any insect bites nor skin lesions in the past, though a habit of oral breathing while sleeping was mentioned. No dermal scars or lesions were found in a physical examination. Diagnostic confirmation and species identification was carried out by polymerase chain reaction (PCR) amplification of the internal transcribed spacer 1 region (ITS1) followed by restriction fragment length polymorphism (RFLP) analysis indicating that the parasite belonged to the *L. donovani* complex (*L. infantum* or *L. donovani*). To differentiate between these species, we used the short cysteine protease E/F - PCR. The amplified product was electrophoresed on 2% agarose gel that generated a 400 bp amplicon typical of *L. donovani* [7]. Given the unusual presentation and patient mild splenomegaly, a serology test was carried out for anti-rK39 antibodies (Kalazar *Detect™* Rapid Test) and found negative. A fourth generation HIV-1/2 antigen/antibody test was negative as well. A diagnosis of Mucosal leishmaniasis (ML) was established and the patient was treated with intravenous liposomal amphotericin B, according to the 2016 Infectious Diseases Society of America (IDSA) and the American Society of Tropical medicine and Hygiene (ASTMH) guidelines, to achieve a cumulative dose of 30 mg/kg body weight [1]. The treatment resulted in marked improvement and complete regression of mucosal lesions by 2-months.

## Discussion and conclusions

This case represents a clinically and laboratory diagnostic challenge. Since the patient resides in an area of Israel endemic for CL, we expected the causative agent to be either *L. tropica* or *L. major*. While *L. infantum* has been reported to cause ML in other regions of the Mediterranean basin, leishmaniasis caused by *L. donovani* is rare in Israel and primarily found in East African refugees with HIV-VL co-infections. Therefore, although described, it was surprising for us to identify the parasites causing ML as *L. donovani* 4 years following a stay in central and North Africa with no evidence or report of former skin lesions. The fact that visceralizing *Leishmania* can produce mucosal disease, raises the question whether the lesions observed in this patient represent the primary site of parasite inoculation (as this patient is reported to breath orally during sleep), or a secondary localization following current or former VL, especially in the presence of an enlarged spleen. The absence of other clinical manifestations of VL including fever, pancytopenia and enlarged liver, as well as the lack of a serological response, do not support concurrent VL. It is worth noting that serology with an rK39-based immunochromatographic test may provide supportive evidence for a diagnosis of VL (specifity 91%), but it is not recommended as a stand-alone VL diagnostic test; however, it may be useful to direct more invasive testing [1]. The rK39-test sensitivity for diagnosing VL in populations unlikely to have HIV/AIDS is considered high (94%) [1], and lowers the likelihood of VL in the present case. Nevertheless, the cumulative dose of liposomal amphotericin B given to the patient (30 mg/kg) was also compatible with the recommended dose for treating VL (21 mg/kg if immunocompetent) [1], and other etiologies causing enlarged spleen should be further searched following repeated ultrasonography.

The dense infiltrate of inflammatory cells with abundant *Leismania* organisms found in histopathological sections in this case are not typical to ML. The key features of ML are delayed type hypersensitivity manifesting as abundant granulomas and low number of parasites in the lesions, mediated by an exaggerated Th1 response with elevated IFN-γ and TNF-α production and lower IL-10 [8]. Interestingly, *Leishmania* RNA Virus 1 (LRV1) harbored by *L. brazilensis* and L. guyanensis was found to induce proinflammatory cytokines and chemokines that contribute to autophagy, tissue destruction and disease exacerbation in ML [9]. It is possible that Old world *Leishmania* species that cause ML, trigger a different immune response that led to abundant parasites in lesions and diminished formation of granulomas, or that a genetic driven local or systemic immunosuppression in this patient resulted in atypical immunopathology [10].

In conclusion, we present a case of *L. donovani* ML in an Israeli resident with a former travel to central and North Africa, with no documented or prior cutaneous lesions. A possible explanation for the mucosal presentation could have been former asymptomatic or subclinical VL with hematogenous dissemination to mucosal membranes, but this hypothesis was not supported by the sensitive rk39 dipstick test. Therefore, though uncommon, ML in this patient probably presents the only manifestation of *L. donovani* infection. A delay in diagnosis and treatment, such as in this case, have led to progression of the maxillary gingival lesions towards the hard palatal and the soft palate, and could have potentially compromised the upper airway. This underscores the importance of early searching for the presence of *Leishmania* in patients with suspected neoplasia or autoimmune disease of the upper airway or oral cavity, if they have resided in *Leishmania* endemic zones. Laboratory processing and molecular workup are necessary to appropriately characterize and treat the disease.

## Abbreviations

ML: Mucosal leishmaniasis
CL: Cutaneous leishmaniasis
VL: Visceral leishmaniasis
